# Genetic Analysis of Persistency for Milk Fat Yield in Iranian Buffaloes (*Bubalus bubalis*)

**DOI:** 10.3389/fgene.2021.633017

**Published:** 2021-03-08

**Authors:** Mohammad Ali Nazari, Navid Ghavi Hossein-Zadeh, Abdol Ahad Shadparvar, Davood Kianzad

**Affiliations:** ^1^Department of Animal Science, Faculty of Agricultural Sciences, University of Guilan, Rasht, Iran; ^2^Animal Breeding Center and Promotion of Animal Products, Karaj, Iran

**Keywords:** dairy buffalo, genetic parameter, genetic trend, lactation persistency, random regression model

## Abstract

This study aimed to estimate heritabilities and genetic trends for different persistency measures for milk fat yield and their genetic correlations with 270-day milk yield in Iranian buffaloes. The records of test-day milk fat yield belonging to the first three lactations of buffaloes within 523 herds consisting of 43,818 records were got from the Animal Breeding Center and Promotion of Animal Products of Iran from 1996 to 2012. To fit the lactation curves based on a random regression test-day model, different orders of Legendre polynomial (LP) functions were selected. Three persistency measures were altered according to the specific condition of the lactation curve in buffaloes: (1) The average of estimated breeding values (EBVs) for test day fat yield from day 226 to day 270 as a deviation from the average of EBVs from day 44 to day 62 (PM_1_), (2) A summation of contribution for each day from day 53 to day 247 as a deviation from day 248 (PM_2_), and (3) The difference between EBVs for day 257 and day 80 (PM_3_). The estimates of heritability for PM_1_, PM_2_, and PM_3_ ranged from 0.20 to 0.48, from 0.36 to 0.47, and from 0.19 to 0.35 over the first three lactations, respectively. The estimate of genetic trends for different persistency measures of milk fat yield was not significant over the lactations (*P > 0*.05). Genetic correlation estimates between various measures of persistency were generally high over the first three lactations. Also, genetic correlations estimates between persistency measures and 270-day milk yield were mostly low and varied from 0.00 to 0.24 (between PM_1_ and 270-day milk yield), from −0.19 to 0.13 (between PM_2_ and 270-day milk yield), and from −0.02 to 0.00 (between PM_1_ and 270-day milk yield) over the first three lactations, respectively. Persistency measures that showed low genetic correlations with milk fat yield were considered the most suitable measures in selection schemes. Besides, medium to high heritability estimates for different persistency measures for milk fat yield indicated that relevant genetic variations detected for these characters could be regarded in outlining later genetic improvement programs of Iranian buffaloes.

## Introduction

One important step for reaching self-sufficiency in any country is to identify the productive potential of native breeds of animals. The great adaptability of native animals to harsh conditions such as high environmental humidity and temperature, irregular rainfall, the incidence of different diseases, weak management practices, and low quality of feeds causes native buffaloes of Iran to play an important role in supplying milk and meat as major protein sources. Currently, many Asian countries depend mainly on buffalo as a source of milk and dairy products, especially in rural areas (Safari et al., 2018).

One of the main factors in determining the total milk production over a lactation period is persistency (Muir, 2004). Persistency is defined as the potential of an animal to maintain milk yield at a high extent after reaching the peak of production. The other definition of persistency is the gradual decrease of daily milk production after reaching the peak of the lactation curve (Togashi and Lin, 2004). The major cause for the worth of buffaloes with more persistent curves is that they can relatively satisfy most parts of their nutrient requirements from roughages (Sölkner and Fuchs, 1987). Therefore, not only metabolic problems, reproduction disorders, and diseases are lower in cows with more persistent lactations, but also production costs would be lower (Dekkers et al., 1998). Determining the method of measuring persistency is a critical point in estimating genetic progress for this trait. However, no general agreement is existent on the most appropriate method to describe the persistency of lactation (Cole and VanRaden, 2006). Various measures were suggested for calculating persistency (Gengler, 1996): measures based on the functions describing persistency; measures based on a fraction of total yield, peak yield, or parts of lactation; and those based on the breeding value of animals derived from analyzing random regression models.

The method used for defining persistency measures would determine the genetic parameter estimates for these measures and their genetic relationship with milk production (Swalve and Gengler, 1999; Jakobsen et al., 2002; Khorshidie et al., 2012). A measure of persistency must have two characteristics: association with lactation curve flatness, and independent explanation from production level. The latter item implies that the genetic correlation between milk yield and persistency measures to be decreased because milk production explains some genetic variance of persistency measures under study (Muir, 2004; Cole and VanRaden, 2006; Khorshidie et al., 2012). The independence of these two traits causes genetic selection for persistency of lactation and total yield to avoid unfavorable consequences of peak yield stress in high-yielding cows. Also, the incidence of metabolic diseases and reproductive disorders would be minimized while high milk production is maintained (Elmaghraby, 2012; Ghavi Hossein-Zadeh et al., 2017).

Previous studies carried out on dairy cattle indicated that lactation persistency positively correlated with favorable reproductive performance and health status (Jakobsen et al., 2002; Muir, 2004). Such favorable correlations along with the positive economic value for persistency would support including lactation persistency in the genetic improvement programs of cattle and buffalo (Dekkers et al., 1998; Khorshidie et al., 2012; Ghavi Hossein-Zadeh et al., 2017).

The random regression models enable fitting random genetic and environmental effects at different stages of lactation, which results in higher accuracy of estimated breeding values (EBVs) compared with other statistical models (Li et al., 2020). These models provide insights about the temporal variation of biological processes and physiological implications underlying the studied traits. Therefore, random regression models generate relevant information to be exploited in breeding programs (Oliveira et al., 2019). The functions generally used to model the lactation curve include Wood’s model (Wood, 1967), Wilmink’s function (Wilmink, 1987), spline function (White et al., 1999), and Legendre polynomial (LP) function (Kirkpatrick et al., 1990). Because of variations in production environments and management systems, optimal functions for test-day models in various countries may be different (Mrode et al., 2003). But several studies have indicated that LPs performed well in random regression test-day models (Li et al., 2020).

Milk constituents can be used as a simple indicator of the nutritional status of the lactating animals. Because of the dilution effect, milk fat percentage shows the opposite direction of the lactation curve for milk yield (Eicher, 2004; Ghavi Hossein-Zadeh, 2016), but fat yield follows a variation trend similar to milk yield over the lactation. When trying to apply milk composition as a nutritional evaluation tool, these fluctuations should be noticed. Although several researchers have studied the genetic analysis of the persistency for milk yield and components in dairy cattle (Cole and Null, 2009; Khorshidie et al., 2012; Canaza-Cayo et al., 2015), limited studies have been performed to estimate genetic parameters of persistency for milk production traits in buffaloes (Ghavi Hossein-Zadeh et al., 2017). Therefore, the objective of the present study was to estimate the heritability and genetic trend of distinct persistency measures for milk fat yield and their genetic correlations with 270-day milk yield in Iranian buffaloes using random regression test-day models.

## Materials and Methods

### Data

Records of test-day milk fat yield belonging to the first three lactations of Iranian native buffaloes in 523 herds consisting of 43,818 records were provided by the Animal Breeding Center and Promotion of Animal Products of Iran during 1996–2012. According to climatic conditions, Iranian native buffaloes can be grouped into three main classes: Azari ecotype, Kuhzestani ecotype, and Mazandarani or North ecotype (Ghavi Hossein-Zadeh et al., 2012). Borghese (2005) and Ghavi Hossein-Zadeh (2015a, b) described the overall management practices and population structure of buffaloes in Iran. Outliers that appeared to deviate markedly from other observations in the original data set were discarded. Therefore, the subsequent analyses included only production records corresponding to the first three lactations in which days in milk (DIM) were between 5 and 270. Calving ages ranged between 24–60, 39–76, and 54–100 months for the first, second, and third lactations, respectively. The total number of test-day records per animal was from 4 to 9. Summary statistics of the edited data set are presented in Table 1. The number of animals, sires, and dams in the pedigree of Iranian buffaloes was 42,285, 549, and 6,376, respectively.

**TABLE 1:** Summary statistics of edited milk fat yield data used in this study.

Days in milk classes	Lactation 1			Lactation 2			Lactation 3		
	N	Mean (kg)	SD (kg)	N	Mean (kg)	SD (kg)	N	Mean (kg)	SD (kg)
5–30	756	0.432	0.225	686	0.461	0.251	654	0.487	0.252
31–60	943	0.426	0.225	956	0.464	0.247	859	0.487	0.252
61–90	1,095	0.488	0.243	985	0.473	0.249	989	0.499	0.257
91–120	1,252	0.477	0.251	1,071	0.492	0.257	1,033	0.508	0.256
121–150	1,176	0.487	0.252	1,013	0.497	0.254	945	0.500	0.263
151–180	1,156	0.474	0.252	1,028	0.489	0.261	906	0.481	0.256
181–210	1,014	0.466	0.252	783	0.480	0.253	711	0.450	0.245
211–240	806	0.444	0.245	611	0.463	0.244	592	0.462	0.255
241–270	569	0.469	0.246	455	0.459	0.244	420	0.433	0.235

### Statistical and Genetic Analysis

Legendre polynomial functions were chosen to fit the lactation curves in the framework of a random regression test-day model for estimating (co)variance components. Model specification and the choice of fixed effects to be included in the model were based on the backward elimination method and variables which were significant at *P* < 0.05 were considered in the model. To obtain the appropriate random regression test-day model for the genetic analysis of test day fat yield, with the minimum number of parameters, different orders of fit for random regression coefficients of additive genetic and permanent environmental effects were evaluated. Also, the optimum set of polynomials was selected according to the logarithm of the likelihood function at the point of conversion and the total number of parameters to be estimated. The difference of these models was based on the LPs applied to fit the covariance functions for additive genetic and permanent environmental effects. The maximum logarithm likelihood of the models was compared and models with the lowest values of this criterion were selected for further analysis. Test day records were analyzed using the following random regression model:

$$Y_{\mathrm{ijmnptv}}=G_{i}+{\mathrm{YS}}_{j}+{\mathrm{HTD}}_{m}+\sum_{f=0}^{2}c_{f}\,{\left({\mathrm{age}}_{n}\right)}^{f}+\sum_{r=0}^{k}\beta_{r}\,\emptyset_{r}\,\left(\dim_{t}\right)+\sum_{r=0}^{{\text{k}}_{a-1}}\alpha_{\text{pr}}\,\emptyset_{\text{r}}\,\left({\text{dim}}_{\text{t}}\right)+\sum_{r=0}^{{\text{k}}_{p-1}}\gamma_{\text{pr}}\,\emptyset_{\text{r}}\,\left({\text{dim}}_{\text{t}}\right)+e_{\text{ijmnptv}}$$

Where,

Y_ijmnptv_ : test day record *i* obtained at DIM *t* of cow *p* calved at the *n*^*th*^ age in herd-test date m,

G_*i*_ : fixed effect of *i*^*th*^ breed or ecotype,

YS_j_ : fixed effect of *j*^*th*^ calving year-season,

HTD_*m*_: fixed effect of *m*^*th*^ herd-test date,

c_*f*_: the f^*th*^ fixed regression coefficient for calving age,

age_*n*_: the *n*^*th*^ calving age,

*k*: the order of fit for fixed regression coefficients (*k* = 2),

β_r_: the *r*^*th*^ fixed regression coefficient,

*k*_*a*_: the order of fit for additive genetic random regression coefficients,

*k*_*p*_: the order of fit for permanent environmental random regression coefficients,

α_*pr*_: the *r*^*th*^ random regression coefficient of additive genetic value for *p*^*th*^ cow,

γ_pr_: the *r*^*th*^ random regression coefficient of permanent environmental effect for *p*^*th*^ cow,

∅_r_(dim_t_): the *r*^*th*^ coefficient of LPs evaluated at days in milk *t*,

e_ijmnptv_: the random residual error.

All random regression analyses were conducted using the Average Information Restricted Maximum Likelihood (AIREML) algorithm of the WOMBAT program (Meyer, 2006).

### Lactation Persistency Measures

The following measures were used to describe lactation persistency in this study. These measures were modified based on the lactation curve conditions of buffaloes and adapted for 270 days lactation period:

1. The average of EBVs for test day fat yield from day 226 to day 270 as a deviation from the average of EBVs from day 44 to day 62 [adapted from Kistemaker (2003)]:

$$P\,M_{1}=\frac{1}{44}\,\sum_{\text{i}=226}^{270}{\text{EBV}}_{\text{i}}-\frac{1}{21}\,\sum_{\text{i}=44}^{62}{\text{EBV}}_{\text{i}}$$

2. A summation of contribution for each day from day 53 to day 247 as a deviation from day 248 [adapted from Cobuci et al. (2007) and Jakobsen et al. (2002)]:

$$P\,M_{2}=\sum_{\text{i}=53}^{247}\left({\text{EBV}}_{\text{i}}-{\text{EBV}}_{\,248}\right)$$

3. The difference between EBVs for day 257 and day 80 [adapted from Cobuci et al. (2004, 2007)]:

$$P\,M_{3}=\left({\text{EBV}}_{257}-{\text{EBV}}_{\,80}\right)$$

Small absolute values of the abovementioned measures indicate a high lactation persistency. If ${\hat{\alpha }}_{i}$ was a (k_*a*_×1) vector of the estimates of additive genetic random regression coefficients specific to the animal *i*, and *Z*_*t*_ was a (*k*_*a*_×1) vector of LP coefficients evaluated at day *t*, the EBV of animal *i* for day *t* was calculated as follows:

$$E\,B\,V_{\text{a}}=\,\sum_{\text{i}=0}^{\text{ka}-1}{\text{a}}_{\text{ij}}\,\,\emptyset_{\text{j}}\,\,\left({\text{dim}}_{\text{t}}\right)={\hat{a}}_{0\,i}\,\emptyset_{0\,t}+{\hat{a}}_{1\,i}\,\emptyset_{1\,t}+{\hat{a}}_{2\,i}\,\emptyset_{2\,t}+{\hat{a}}_{3\,i}\,\emptyset_{3\,t}$$

Therefore, the EBV of animal i for 270-day production was obtained by summing the EBVs from day 5 to day 270:

$$E\,B\,V\,T_{i}=\sum_{5}^{270}\left({\hat{a}}_{0\,i}\,\emptyset_{0\,i}\,{\hat{a}}_{1\,i}\,\emptyset_{1\,i}\,{\hat{a}}_{2\,i}\,\emptyset_{2\,i}\,{\hat{a}}_{3\,i}\,\emptyset_{3\,i}\right)=\left(\sum_{5}^{270}\emptyset_{0\,t}\,\sum_{5}^{270}\emptyset_{0\,t}\,\sum_{5}^{270}\emptyset_{0\,t}\,\sum_{5}^{270}\emptyset_{0\,t}\right)\,{\hat{a}}_{i}=Z_{c\,270}\,{\hat{\text{a}}}_{\text{i}}$$

Where, *Z*_*c270*_ is a vector of the summations of LPs corresponding to total lactation yield. In addition to the 270-day yield, we could estimate a *Z_c* corresponding to each persistency measures used in the current study as follows:

For the first lactation fat yield:

$$\begin{array}{ccccc} \mathrm{Zc}{}_{270} & = & (0.7071\; & 1.42\,\text{E}-17\; & 0.0059)\\ \mathrm{ZcP}{}_{1\,g} & = & (0\; & 0.7839\; & 0.8491)\\ \mathrm{ZcP}{}_{2\,g} & = & (0\; & 1.6361\; & 1.4825)\\ \mathrm{ZcP}{}_{3\,g} & = & (0\; & -0.9058\; & -1.2003) \end{array}$$

For second lactation fat yield:

$$\begin{array}{ccccccc} \mathrm{Zc}{}_{270} & = & (0.7071\; & 1.42\,\text{E}-17\; & 0.0059\; & -6.7\,\text{E}-18\; & 0.0081)\\ \mathrm{ZcP}{}_{1\,g} & = & (0\; & 0.7839\; & 0.8491\; & -0.0664\; & 0.9943)\\ \mathrm{ZcP}{}_{2\,g} & = & (0\; & 1.6361\; & 1.4825\; & 0.0645\; & 0.8387)\\ \mathrm{ZcP}{}_{3\,g} & = & (0\; & -0.9058\; & -1.2003\; & -0.3943\; & 0.1711) \end{array}$$

For third lactation fat yield:

$$\begin{array}{ccccccc} \mathrm{Zc}{}_{270} & = & (0.7071\; & 1.42\,\text{E}-17\; & 0.0059\; & -6.7\,\text{E}-18\; & 0.0081)\\ \mathrm{ZcP}{}_{1\,g} & = & (0\; & 0.7839\; & 0.8491\; & -0.0664\; & 0.9943)\\ \mathrm{ZcP}{}_{2\,g} & = & (0\; & 1.6361\; & 1.4825\; & 0.0645\; & 0.8387)\\ \mathrm{ZcP}{}_{3\,g} & = & (0\; & -0.9058\; & -1.2003\; & -0.3943\; & 0.1711) \end{array}$$

### Estimation of Genetic Parameters and Genetic Trends

The following formulas were applied to estimate additive genetic, permanent environmental and residual variances and heritabilities for different measures of persistency for fat yield and 270-day milk yield:

σa(pi,E⁢B⁢V270⁢M⁢Y)=Zcpi⁢g⁢Ka⁢Zc270⁢M⁢Y⁢g′σp⁢epi2=Zcpi⁢p⁢e⁢Kp⁢e⁢Zcpi⁢p⁢e′hpi2=σapi2σp⁢hpi2σa270⁢M⁢Y2=Zc270⁢M⁢Y⁢g⁢Ka⁢Zc270⁢M⁢Y⁢g′σp⁢e270⁢M⁢Y2=Zc270⁢M⁢Y⁢p⁢e⁢Ka⁢Zc270⁢M⁢Y⁢p⁢e′

$$\begin{array}{c} \sigma_{e}^{2}=8.85\,K\,g^{2}\\ \sigma_{e_{p_{1}}}^{2}=\left(\frac{1}{44}+\frac{1}{18}\right)\,\sigma_{e}^{2}\\ \sigma_{e_{p_{2}}}^{2}=48620\,\sigma_{e}^{2}\\ \sigma_{e_{p_{3}}}^{2}=2\,\sigma_{e}^{2}\\ \sigma_{e_{270\,M\,Y}}^{2}=266\,\sigma_{e}^{2} \end{array}$$

Where, *K*_a_ and *K*_pe_ are matrices of direct additive genetic and permanent environmental (co)variances of random regression coefficients, $\sigma_{a_{p_{i}}}^{2}$, $\sigma_{p\,e_{p_{i}}}^{2}$, $\sigma_{p\,h_{p_{i}}}^{2}$, and $h_{p_{i}}^{2}$ are the additive genetic, permanent environmental, phenotypic variances, and heritability estimate for i^*th*^ persistency measure and $\sigma_{a_{270\,M\,Y}}^{2}$, $\sigma_{p\,e_{270\,M\,Y}}^{2}$, $\sigma_{p\,h_{270\,M\,Y}}^{2}$, and $h_{270\,M\,Y}^{2}$ are the additive genetic, permanent environmental, phenotypic variances, and heritability estimate for 270-day milk yield, respectively. $\sigma_{e}^{2}$ is a constant residual variance estimated for each day of lactation and $\sigma_{e_{p_{1}}}^{2}$, $\sigma_{e_{p_{2}}}^{2}$, $\sigma_{e_{p_{3}}}^{2}$, and $\sigma_{e_{270\,M\,Y}}^{2}$ are residual variances for persistency measures PM_1_, PM_2_, PM_3_, and 270-day milk yield, respectively. Also, phenotypic variances were obtained by summing the genetic, permanent environmental, and residual variances for different persistency measures and milk yield. Estimates of genetic correlations among persistency measures and with 270-day milk yield were obtained as follows:

σa(pi,pj)=Zcpi⁢g⁢Ka⁢Zcpj⁢g′

σa(pi,E⁢B⁢V270⁢M⁢Y)=Zcpi⁢g⁢Ka⁢Zc270⁢M⁢Y⁢g′

$$R_{a_{\left(p_{i},p_{j}\right)}}=\frac{\sigma_{a_{\left(p_{i},p_{j}\right)}}}{\sqrt{\left(\sigma_{a_{p_{i}}}^{2}\right)\,\left(\sigma_{a_{p_{j}}}^{2}\right)}}$$

$$R_{a_{\left(p_{i},E\,B\,V_{270\,M\,Y}\right)}}=\frac{\sigma_{a_{\left(p_{i},E\,B\,V_{270\,M\,Y}\right)}}}{\sqrt{\left(\sigma_{a_{p_{i}}}^{2}\right)\,\left(\sigma_{a_{270\,M\,Y}}^{2}\right)}}$$

Where, σ_*a*__(*pi*,*pj*)_, σ_*a*__(*pi*,*EBV*_270*MY*_)_, *R*_*a*__(*pi*,*pj*)_, and *R*_*a*__(*pi*,*EBV*__270*MY*_) are genetic covariances and correlations between persistency measures and 270-day milk yield, respectively. Estimates of genetic trends for persistency measures were obtained by regressing the average EBVs on the calving year of animals.

## Results

The orders of fit for different random regression test-day models of milk fat production are given in Table 2. The maximum log-likelihood values of test-day models 1, 10, and 10 differed significantly (*P* < 0.05) from the other models for fat yield in the first three lactations, respectively. Thus, models 1, 10, and 10 were chosen to fit the additive genetic and permanent environmental effects for the analysis of fat production in the first three lactations of buffaloes, respectively.

**TABLE 2:** Orders of fit for different random regression test-day models of milk fat yield evaluated in this study.

Model	Order of fit		NP^3^	Maximum log-likelihood		
	k_*a*_^1^	k_*pe*_^2^		Lactation 1	Lactation 2	Lactation 3
1	3	3	21	−5,593.84*	−7,188.50	−6,996.65
2	3	4	25	−5,478.18	−7,187.89	−7,021.78
3	3	5	30	−5,486.11	−7,190.54	−7,030.21
4	3	6	36	−5,487.47	−7,194.83	−7,046.79
5	4	3	25	−5,577.40	−7,190.17	−7,026.02
6	4	4	29	−5,483.49	−7,191.63	−7,025.94
7	4	5	34	−5,482.32	−7,192.05	−7,026.09
8	4	6	40	−5,492.64	−7,198.73	−7,049.79
9	5	5	39	−5,495.48	−7,200.18	−7,041.73
10	5	6	45	−5,495.71	−7,204.1*	−7,050.93*

^1^k_*a*_ = orders of fit for additive genetic effects. ^2^k_*pe*_ = orders of fit for permanent environmental effects. ^3^NP: number of the parameter for estimated variance function. *Significant at *P* < 0.05.

Heritability estimates of persistency measures for fat production and estimates of genetic correlation among distinct fat yield persistency measures with each other and with 270-day milk production in Iranian buffaloes are presented in Table 3. Heritability estimates for PM_1_, PM_2_, and PM_3_ ranged between 0.20–0.48, 0.36–0.47, and 0.19–0.35 for the first, second, and third lactations, respectively. In general, heritability estimates fluctuated largely among lactations and persistency measures. The highest estimate of heritability was observed for PM_1_ in the third lactation (0.48), while the lowest one was recorded for PM_3_ also in the third lactation.

**TABLE 3:** Heritability estimates of different persistency measures for milk fat yield and genetic correlations among distinct fat yield persistency measures with each other and with 270-day milk production in Iranian buffaloes.

Trait	Lactation 1	Lactation 2	Lactation 3
	
	Heritability		
PM_1_	0.20	0.39	0.48
PM_2_	0.47	0.36	0.46
PM_3_	0.31	0.35	0.19

		**Genetic correlation**	

PM_1_-PM_2_	0.99	0.98	0.98
PM_1_-PM_3_	−0.98	–0.90	−0.87
PM_2_-PM_3_	−0.99	–0.95	−0.95
PM_1_-270 d MY	0.05	0.00	0.24
PM_2_-270 d MY	0.01	–0.19	0.13
PM_3_-270 d MY	0.00	0.00	−0.02

*PM_1_, the average EBVs for test day milk fat yield from day 226 to day 270 as a deviation from the average of EBVs from day 44 to day 62; PM_2_, a summation of contribution for each day from day 53 to day 247 as a deviation from day 248; PM_3_, The difference between EBVs for day 257 and day 80; 270 d MY, 270-day milk production.*

Genetic correlation estimates among various measures of persistency were generally high and ranged from 0.98 to 0.99 (between PM_1_ and PM_2_), from −0.98 to −0.87 (between PM_1_ and PM_3_), and from −0.99 to −0.95 (between PM_2_ and PM_3_) over the first three lactations, respectively. Also, genetic correlation estimates between persistency measures and milk yield were mostly low and varied from 0.00 to 0.24 (between PM_1_ and 270-day milk yield), from −0.19 to 0.13 (between PM_2_ and 270-day milk yield), and from −0.02 to 0.00 (between PM_3_ and 270-day milk yield) across the first three lactations, respectively (Table 3).

**FIGURE 1:**
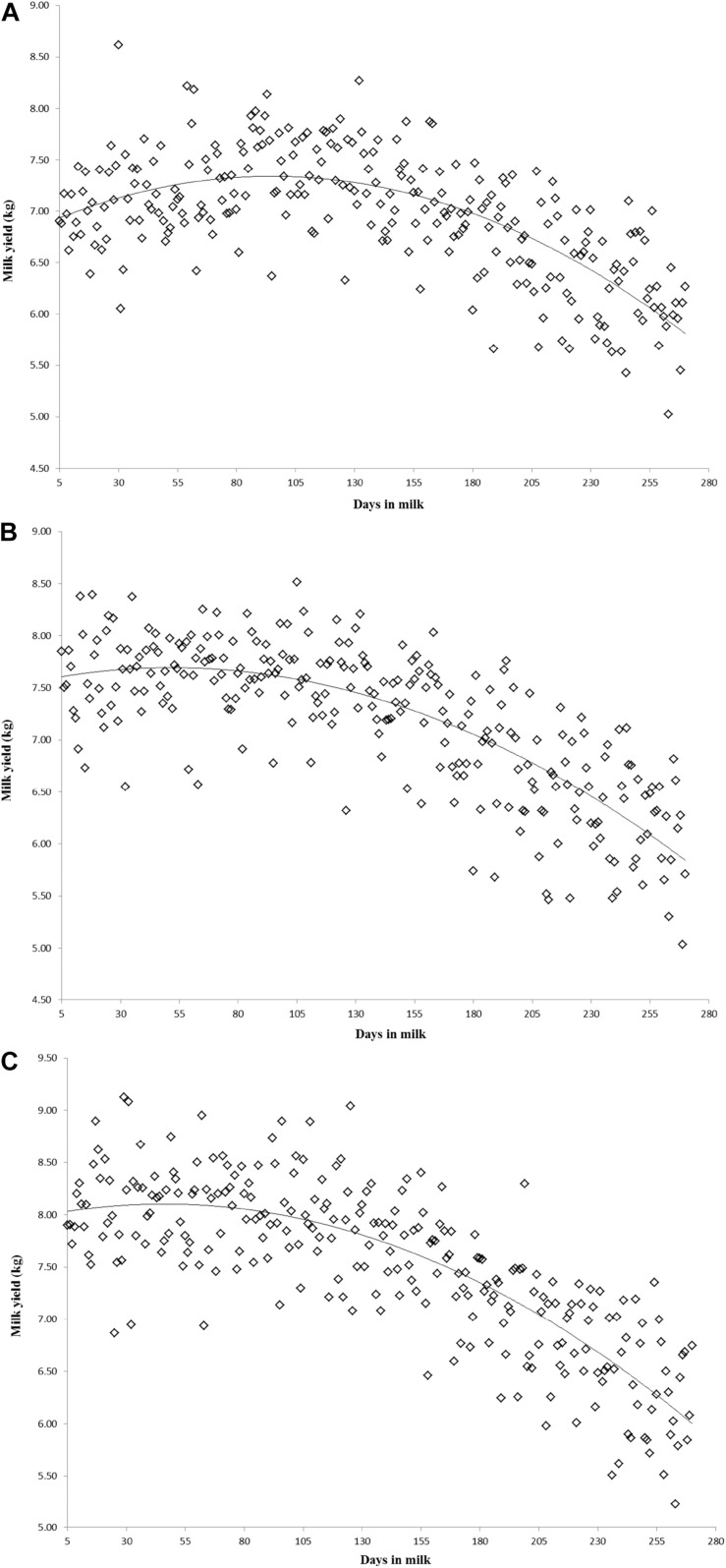
Variation of milk yield across the first **(A)**, second **(B)**, and third **(C)** lactations in Iranian buffaloes.

**FIGURE 2:**
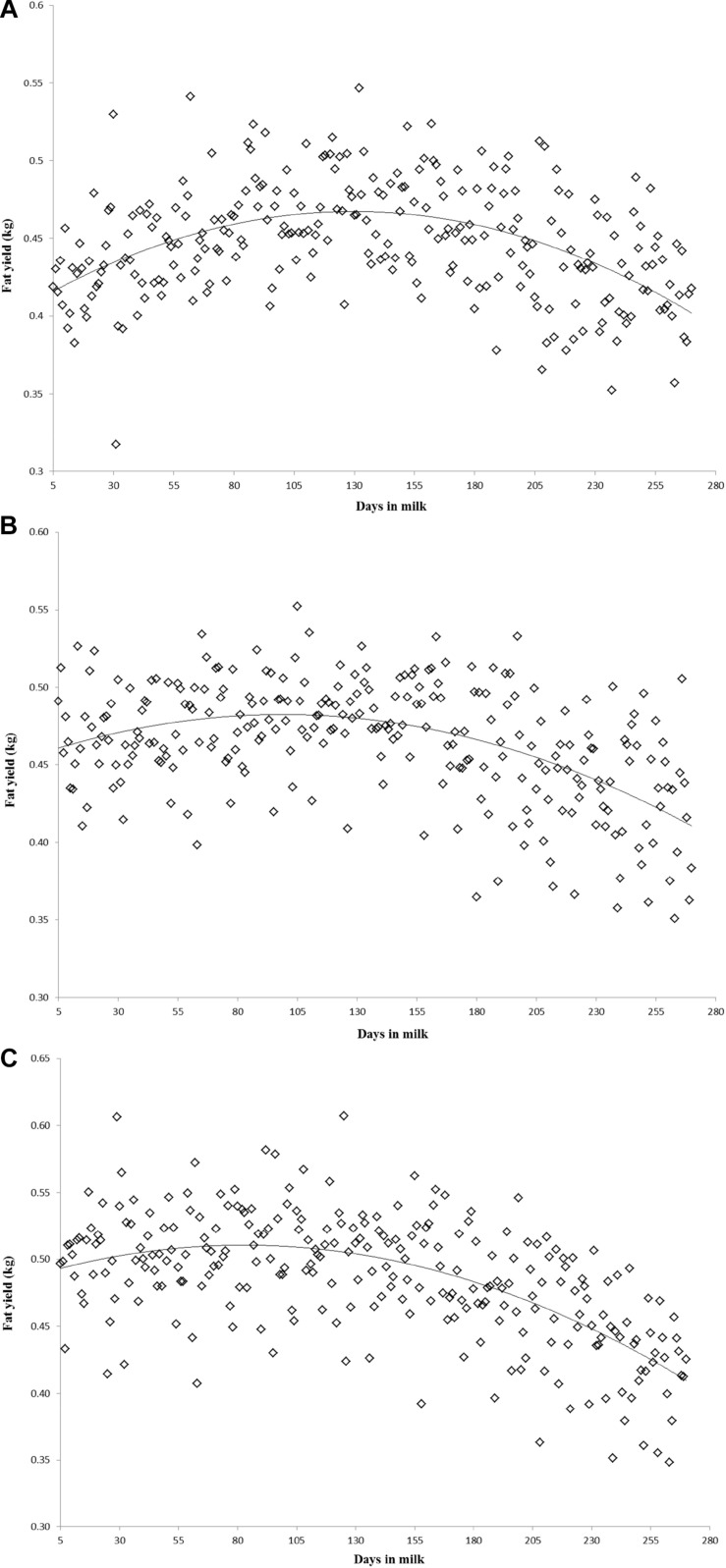
Variation of milk fat yield across the first **(A)**, second **(B)**, and third **(C)** lactations in Iranian buffaloes.

**FIGURE 3:**
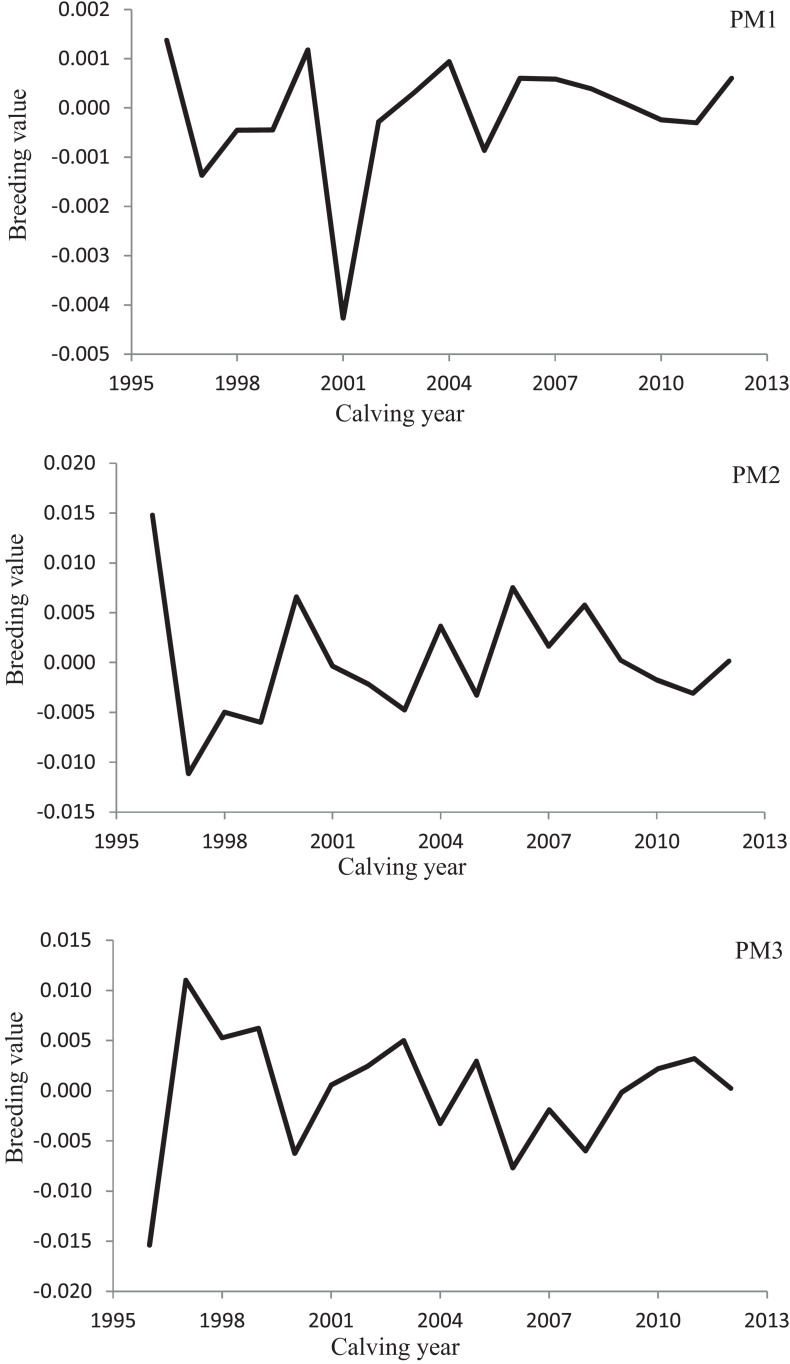
Variation in estimated breeding values of animals for persistency measures of milk fat yield according to calving year in the first lactation.

**FIGURE 4:**
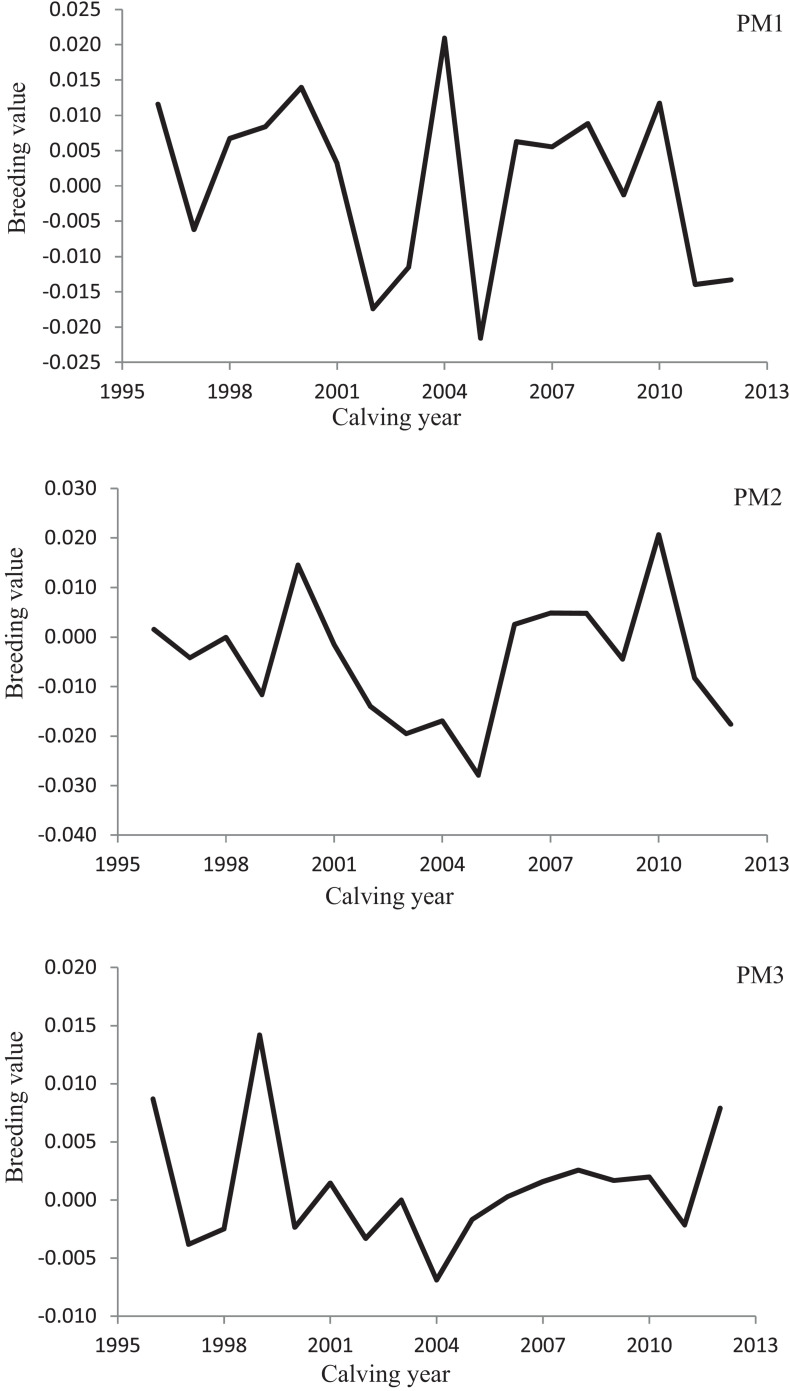
Variation in estimated breeding values of animals for persistency measures of milk fat yield according to calving year in the second lactation.

**FIGURE 5:**
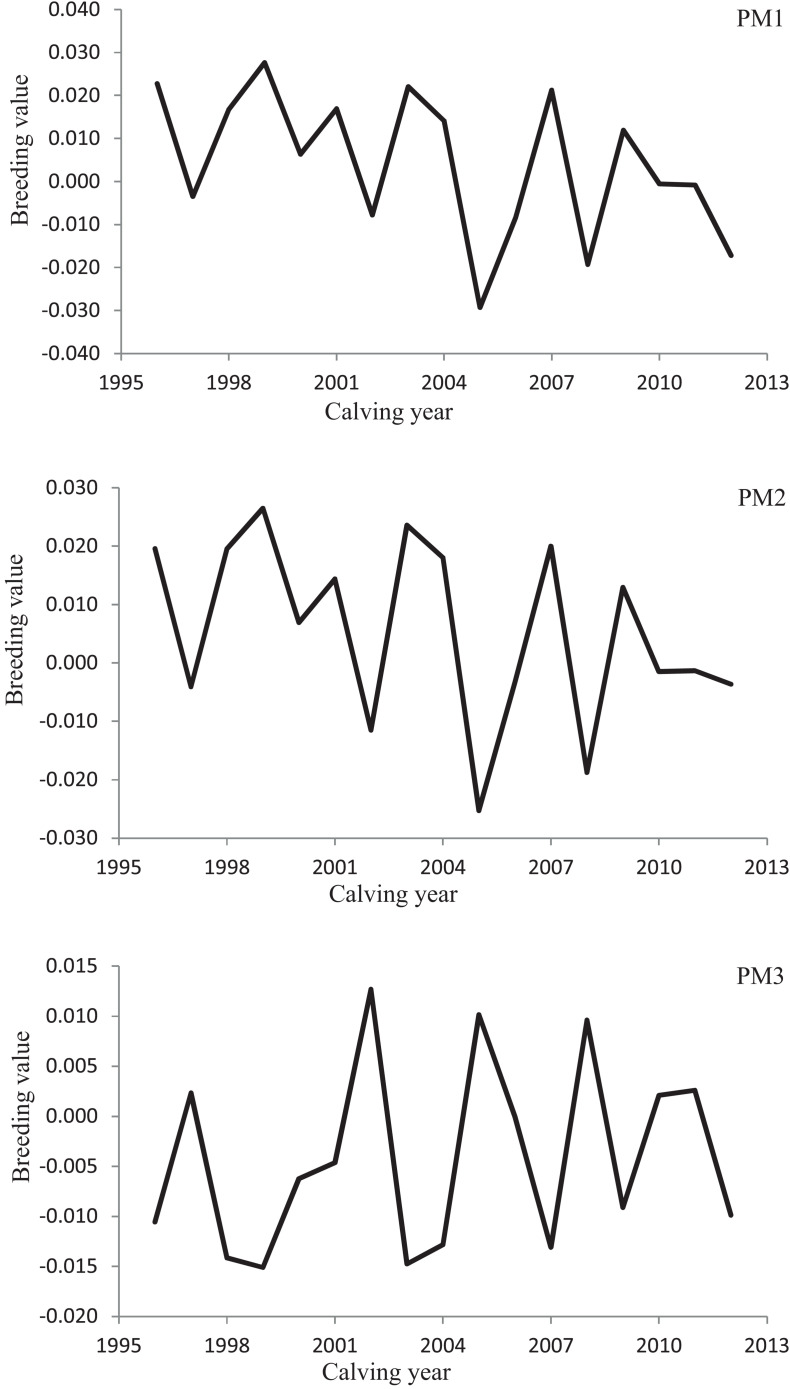
Variation in estimated breeding values of animals for persistency measures of milk fat yield according to calving year in the third lactation.

Variation of milk yield and milk fat yield across the first three lactations of Iranian buffaloes are depicted in Figures 1, 2. The trend of observed milk yield and milk fat yield for all lactations increased from day 5 of lactation to a peak several weeks later, declining thereafter until day 270. Genetic trends of persistency measures for milk fat yield are illustrated in Table 4. In general, all estimates are very low and not significant (*P* > 0.05). Therefore, they would not be considered different from zero. Changes in EBVs of buffaloes for three persistency measures of milk fat yield according to calving year and lactations are illustrated in Figures 3–5. In general, irregular fluctuations were observed in the annual mean predicted breeding values of animals for different persistency measures across the first three lactations.

**TABLE 4:** Estimates of genetic trends for various persistency measures of fat production in buffaloes.

Trait	Lactation 1	Lactation 2	Lactation 3
PM_1_	−0.00004 ± 0.000035	−0.00007 ± 0.00091	−0.0006 ± 0.0008
PM_2_	−0.000013 ± 0.00018	−0.00024 ± 0.00074	−0.0007 ± 0.0008
PM_3_	0.000013 ± 0.00019	−0.000085 ± 0.00026	−0.00052 ± 0.00051

*PM_1_, the average EBVs for test day milk fat production from day 226 to day 270 as a deviation from the average of EBVs from day 44 to day 62; PM_2_, a summation of contribution for each day from day 53 to day 247 as a deviation from day 248; PM_3_, the difference between EBVs for day 257 and day 80.*

## Discussion

For many years, the breeding objectives of dairy animals emphasized increased milk yield. But negative genetic associations were observed between numerous functional characters with production traits, and decreases in genetic excellence for fitness and health have been detected in dairy farms (Egger-Danner et al., 2015). The management practices must be directed toward the compensation of these effects and to equalize reproduction performance, metabolic diseases, and udder health vs. enhanced production to maximize profit without any negative impact on animal welfare. Because concerns on animal welfare and consumers’ appeal for natural and health products are increasing, the functional traits have received greater importance in animal breeding programs (Egger-Danner et al., 2015). In this regard, it is required to have valid genetic parameter estimates for outstanding traits related to the farm profit, including functional traits, in the animal breeding programs (Fleming et al., 2018). Interest to include new traits in the current animal breeding programs is extending to improve simultaneously the production and reproduction performance along with animal health and well-being in dairy farms. Although, for the inclusion of a specific trait into a genetic selection program, it would be inheritable, profitable, quantifiable, and changeable (Wood et al., 2003). Although, there were some reports on the genetic analysis of persistency measures for milk components in dairy cows (Cole and Null, 2009; de Oliveira Biassus et al., 2010), to the knowledge of authors, this is the first report on the genetic analysis of fat production persistency measures in buffaloes.

In general, medium to high heritability estimates for three milk fat persistency measures in this study could be due to the reasonable additive genetic variations for these traits indicating that improvement in these traits could be attained by genetic selection. Regardless of the simpler estimation of PM_3_ in contrast to other measures of persistency, the estimate of heritability for this measure was between the estimates of heritability for PM_1_ and PM_2_ measures for fat yield in the first lactation and had the smallest estimate in second and third parities. If a measure of persistency had higher heritability compared with other measures, this measure would be an appropriate measure to be considered in the selection objective (Ghavi Hossein-Zadeh et al., 2017). Respecting this explanation, the PM_2_ measure would be regarded as the selection criterion in the first lactation, but the measure of PM_1_ would be included as a selection objective in the second-, and third parities. Although there is no report of genetic parameters for persistency measures of fat yield in buffaloes, Cole and Null (2009) reported the estimates of heritability for fat production persistency measure varied from 0.07 to 0.12 in five breeds of dairy cows. Also, de Oliveira Biassus et al. (2010) reported the heritability estimates for fat yield persistency measures ranged from 0.00 to 0.23 in primiparous Holstein cows. Besides, Gengler (1995) estimated the heritability for fat yield persistency measure would be equal to 0.06 in dairy cows. In general, several factors could influence the variation in heritability estimates for milk fat yield persistency obtained in different studies, including the breed of the animal, within-population genetic diversity, management procedures, environmental conditions, and methods used for estimating genetic parameters. According to de Oliveira Biassus et al. (2010), different factors would influence the variations of heritability estimates for persistency measures among studies: the definition of persistency measure as absolute or relative terms, the statistical adequacy of the specific measure of persistency for under study population, the lactation period used to calculate the measure of persistency, and the method or model used to calculate a specific persistency measure. Compared with the first and third lactations, less variation in heritability estimates between the three persistency measures in the second lactation would be due to the differences in lactation curves, yield persistencies, and variation of records across the first three lactations.

The production difference in two different parts of the lactation would be evaluated by the PM_1_ and PM_3_ persistency measures (Ghavi Hossein-Zadeh et al., 2017). Compared with PM_1_ and PM_3_ measures, the PM_2_ measure displayed a domain below the lactation curve at a definite time that has been adjusted for yield at the end section of that period (Khorshidie et al., 2012). The procedure for defining the PM_2_ and PM_3_ persistency measures resulted in a high and negative genetic association between them. The positive genetic correlations between PM_1_ and PM_2_ are proof for the same genetic and physiological systems managing these persistency measures and would cause the same ranking of buffaloes according to these criteria in breeding and genetic schemes (Ghavi Hossein-Zadeh et al., 2017). Contrarily, high negative genetic correlations between PM_3_ with two other persistency measures implied the existence of various mechanisms to govern them. In general, low genetic correlations between different persistency measures for fat yield with milk production point out that selection for a persistency measure for milk fat yield would slightly affect milk yield. In a selection program, it would be favorable to have persistency measures that had low genetic correlations with milk yield (Dekkers et al., 1998; Ghavi Hossein-Zadeh et al., 2017). According to this explanation and regarding the low genetic correlations of persistency measures for fat yield with milk production in the present study, all three measures would be considered as selection criteria that were relatively independent of production level in buffaloes. This finding indicates that a buffalo cow with the highest EBV for 270-day milk yield does not necessarily has the highest EBV for fat yield persistency and vice versa. In the other words, low estimates of genetic association between fat yield persistency measures with milk production signified that buffaloes with the identical quantity of 270-day milk yield could have a distinct extent of persistency across the lactation period (Jamrozik et al., 1998; Cobuci et al., 2007; Ghavi Hossein-Zadeh et al., 2017). The appropriateness of genetic correlation between a specific persistency measure for milk fat yield and milk production depends on the positive or negative mean of the persistency measure in the population under study (Khorshidie et al., 2012). Generally similar to the results of the present study, Cole and Null (2009) observed the estimates of genetic associations between persistency measure of fat yield with 305-day milk production varied from 0.07 to 0.29 in five breeds of dairy cows.

Predicting accurately the animals’ breeding value is an appropriate way to increase the genetic gain in a specific breeding scheme (Ghavi Hossein-Zadeh, 2012). The successfulness of a selection scheme would be assessed by testing the actual alteration in breeding value indicated as a fraction of the expected theoretical modification in the average breeding value of the character under study (Jurado et al., 1994; Ghavi Hossein-Zadeh, 2012). Non-significant genetic progress estimated for all fat production persistency measures in the present study and irregular changes in average EBVs of animals over the years demonstrated the non-presence of a clear breeding design for making better the lactation persistency for fat yield in Iranian buffaloes until now. A possible reason for the non-significant genetic trends of milk fat persistency measures would be the low and close to zero estimates of genetic correlation between fat yield persistency measures and 270-day milk yield in the population under study.

## Conclusion

The persistency measures of fat yield proposed in the present study had favorable low genetic correlations with 270-day milk production. These low correlations would be a benefit in designing a selection program to enhance the milk yield in Iranian buffaloes because buffaloes with the identical quantity of 270-day milk yield could have a distinct extent of persistency across the lactation period. The PM_2_ measure had the highest heritability estimate for the first lactation buffaloes, but the PM_1_ measure had the highest estimate in the second- and -third lactations. Therefore, the PM_2_ measure would be regarded as the selection criterion in the first lactation, but the measure of PM_1_ could be suggested as a selection objective in the second- and third parities. Based on the results of this study, it would be necessary to consider the persistency of fat yield in the selection objective of buffaloes in Iran together with main characters such as production and reproduction traits, and persistency for milk production.

## Data Availability Statement

The data analyzed in this study is subject to the following licenses/restrictions: The data analyzed in this study was obtained from the Animal Breeding Center and Promotion of Animal Products of Iran. Requests to access these datasets should be directed to http://abc.org.ir.

## Ethics Statement

Ethical review and approval was not required for the animal study because datasets used in this study were obtained from pre-existing databases based on routine animal recording procedures.

## Author Contributions

MN participated in the acquisition of data, statistical, and genetic analyses of data. NH-Z designed and conceived this study and contributed to the statistical and genetic analyses of data, and prepared the manuscript. AS contributed to the conception of the study and assisted with the interpretation of the outputs. DK assisted with the interpretation of data. All authors read and approved the final manuscript.

## Conflict of Interest

The authors declare that the research was conducted in the absence of any commercial or financial relationships that could be construed as a potential conflict of interest.

## References

[B1] Borghese A. (2005). *Buffalo Production and Research.’ (FAO Regional Office for Europe Inter-Regional Cooperative Research Network on Buffalo (ESCORENA: Rome, Italy).* Available online at http://www.fao.org/3/ah847e/ah847e.pdf (accessed November 23, 2020).

[B2] Canaza-Cayo A. W. Sávio Lopes P. da Silva M. V. G. B. de Almeida Torres R. Fonseca Martins M. Arbex W. A. (2015). Genetic parameters for milk yield and lactation persistency using random regression models in Girolando cattle. *Asian Australas. J. Anim. Sci.* 28 1407–1418. 10.5713/ajas.14.0620 26323397 PMC4554847

[B3] Cobuci J. A. Euclydes R. F. Costa C. N. Lopes P. S. Torres R. A. Perreira C. S. (2004). Analysis of persistency in the lactation of Holstein cows using test-day yield and random regression model. *Revis. Brasil Zootec.* 33 546–554.

[B4] Cobuci J. A. Euclydes R. F. Costa C. N. Torres R. A. Lopes P. S. Perreira C. S. (2007). Genetic evaluation for persistency of lactation in Holstein cows using a random regression model. *Genet. Mol. Biol.* 30 349–355.

[B5] Cole J. B. Null D. J. (2009). Genetic evaluation of lactation persistency for five breeds of dairy cattle. *J. Dairy Sci.* 92 2248–2258. 10.3168/jds.2008-1825 19389984

[B6] Cole J. B. VanRaden P. M. (2006). Genetic evaluation and best prediction of lactation persistency. *J. Dairy Sci.* 89 2722–2728. 10.3168/jds.S0022-0302(06)72348-7 16772591

[B7] de Oliveira Biassus I. Cobuci J. A. Costa C. N. Rorato P. R. N. Neto J. B. Cardoso L. L. (2010). Persistence in milk, fat and protein production of primiparous Holstein cows by random regression models. *R. Bras. Zootec.* 39 2617–2624. 10.1590/S1516-35982010001200009

[B8] Dekkers J. C. M. ten Hag J. H. Weersink A. (1998). Economic aspects of persistency of lactation in dairy cattle. *Livest. Prod. Sci.* 53 237–252. 10.1016/S0301-6226(97)00124-3

[B9] Egger-Danner C. Cole J. B. Pryce J. E. Gengler N. Heringstad B. Bradley A. (2015). Invited review: overview of new traits and phenotyping strategies in dairy cattle with a focus on functional traits. *Animal* 9 191–207. 10.1017/S1751731114002614 25387784 PMC4299537

[B10] Eicher R. (2004). Evaluation of the metabolic and nutritional situation in dairy herds: diagnostic use of milk components. *Med. Vet. du Quebec.* 34 36–38.

[B11] Elmaghraby M. (2012). Lactation persistency and prediction of total milk yield from monthly yields in Egyptian buffaloes. *Lucrãri ?tiin?ifice* 53 130–137.

[B12] Fleming A. Schenkel F. S. Malchiodi F. Ali R. A. Mallard B. Sargolzaei M. (2018). Genetic correlations of mid-infrared-predicted milk fatty acid groups with milk production traits. *J. Dairy Sci.* 101 4295–4306. 10.3168/jds.2017-14089 29477537

[B13] Gengler N. (1995). Multiple-trait genetic evaluations for milk, fat, and protein yields and persistency. *Interbull Bull.* 11 1–6.

[B14] Gengler N. (1996). Persistency of lactation yields: a review. *Interbull Bull.* 12 87–96.

[B15] Ghavi Hossein-Zadeh N. (2012). Bayesian estimates of genetic changes for body weight traits of Moghani sheep using Gibbs sampling. *Trop. Anim. Health Prod.* 44 531–536. 10.1007/s11250-011-9930-1 21789547

[B16] Ghavi Hossein-Zadeh N. (2015b). Bayesian analysis of direct and maternal effects for birthweight in Iranian buffaloes using Gibbs sampling. *Anim. Prod. Sci.* 56 859–865. 10.1071/AN14564

[B17] Ghavi Hossein-Zadeh N. (2015a). Analysis of population structure and genetic variability in Iranian buffaloes (*Bubalus bubalis*) using pedigree information. *Anim. Prod. Sci.* 56 1130–1135. 10.1071/AN14738

[B18] Ghavi Hossein-Zadeh N. (2016). Modelling lactation curve for milk fat to protein ratio in Iranian buffaloes (*Bubalus bubalis*) using non-linear mixed models. *J. Dairy Res.* 83 334–340. 10.1017/S0022029916000340 27600968

[B19] Ghavi Hossein-Zadeh N. Madad M. Shadparvar A. A. Kianzad D. (2012). An observational analysis of secondary sex ratio, stillbirth and birth weight in Iranian buffaloes (*Bubalus bubalis*). *J. Agric. Sci. Technol.* 14 1477–1484.

[B20] Ghavi Hossein-Zadeh N. Nazari M. A. Shadparvar A. A. (2017). Genetic perspective of milk yield persistency in the first three lactations of Iranian buffaloes (*Bubalus bubalis*). *J. Dairy Res.* 84 434–439. 10.1017/S0022029917000498 28929983

[B21] Jakobsen J. H. Madsen P. Jensen J. Pedersen J. Christensen L. G. Sorensen D. A. (2002). Genetic parameters for milk production and persistency for Danish Holsteins estimated in random regression models using REML. *J. Dairy Sci.* 85 1607–1616. 10.3168/jds.S0022-0302(02)74231-8 12146494

[B22] Jamrozik J. Jansen G. Schaeffer L. R. Liu Z. (1998). Analysis of persistency of lactation calculated from a random regression test day model. *Interbull Bull.* 17 64–69.

[B23] Jurado J. J. Alonso A. Alenda R. (1994). Selection response for growth in Spanish Merino flock. *J. Anim. Sci.* 72 1433–1440. 10.2527/1994.7261433x 8071166

[B24] Khorshidie R. Shadparvar A. A. Ghavi Hossein-Zadeh N. Joezy Shakalgurabi S. (2012). Genetic trends for 305-day milk yield and persistency in Iranian Holsteins. *Livest. Sci.* 144 211–217. 10.1016/j.livsci.2011.11.016

[B25] Kirkpatrick M. Lofsvold D. Bulmer M. (1990). Analysis of the inheritance, selection and evolution of growth trajectories. *Genetics* 124 979–993. 10.1093/genetics/124.4.979 2323560 PMC1203988

[B26] Kistemaker G. J. (2003). Comparison of persistency definitions in random regression test day models. *Interbull Bull.* 30 96–98.

[B27] Li J. Gao H. Madsen P. Li R. Liu W. Bao P. (2020). Impact of the order of Legendre polynomials in random regression model on genetic evaluation for milk yield in dairy cattle population. *Front. Genet.* 11:586155. 10.3389/fgene.2020.586155 33250923 PMC7674963

[B28] Meyer K. (2006). *WOMBAT – A Program for Mixed Model Analyses by Restricted Maximum Likelihood. User Notes.* Armidale: Animal Genetics and Breeding Unit, University of New England.

[B29] Mrode R. A. Swanson G. J. T. Paget M. F. (2003). “Implementation of a test day model for production traits in the UK,” in *Proceedings of the Interbull Meeting*, (Cambridge: Cambridge University Press), 193–196.

[B30] Muir B. L. (2004). *Genetics of Lactation Persistency and Relationships with Reproductive Performance in Holsteins.* Ph.D. Dissertation, University of Guelph, Guelph, ON.

[B31] Oliveira H. R. Brito L. F. Lourenco D. A. L. Silva F. F. Jamrozik J. Schaeffer L. R. (2019). Invited review: advances and applications of random regression models: from quantitative genetics to genomics. *J. Dairy Sci.* 102 7664–7683. 10.3168/jds.2019-16265 31255270

[B32] Safari A. Ghavi Hossein-Zadeh N. Shadparvar A. A. Abdollahi Arpanahi R. (2018). A review on breeding and genetic strategies in Iranian buffaloes (*Bubalus bubalis*). *Trop. Anim. Health Prod.* 50 707–714. 10.1007/s11250-018-1563-1 29524107

[B33] Sölkner J. Fuchs W. (1987). A comparison of different measures of persistency with special respect to variation of test-day milk yields. *Livest. Prod. Sci.* 16 305–319. 10.1016/0301-6226(87)90001-7

[B34] Swalve H. H. Gengler N. (1999). Genetics of lactation persistency. *Occ. Publ. Br. Soc. Anim. Sci.* 24 75–82. 10.1017/S1463981500043090

[B35] Togashi K. Lin C. Y. (2004). Efficiency of different selection criteria for persistency and lactation milk yield. *J. Dairy Sci.* 87 1528–1535. 10.3168/jds.S0022-0302(04)73304-4 15291002

[B36] White I. M. S. Thompson R. Brotherstone S. (1999). Genetic and environmental smoothing of lactation curves with cubic splines. *J. Dairy Sci.* 82 632–638. 10.3168/jds.S0022-0302(99)75277-X 10194684

[B37] Wilmink J. B. M. (1987). Adjustment of test-day milk, fat and protein yield for age, season and stage of lactation. *Livest. Prod. Sci.* 16 335–348. 10.1016/0301-6226(87)90003-0

[B38] Wood G. M. Boettcher P. J. Jamrozik J. Jansen G. B. Kelton D. F. (2003). Estimation of genetic parameters for concentrations of milk urea nitrogen. *J. Dairy Sci.* 86 2462–2469. 10.3168/jds.S0022-0302(03)73840-5 12906064

[B39] Wood P. (1967). Algebraic model of the lactation curve in cattle. *Nature* 216:164. 10.1038/216164a0

